# A quantitative imaging framework for lithium morphology: Linking deposition uniformity to cycle stability in lithium metal batteries

**DOI:** 10.1073/pnas.2502518122

**Published:** 2025-07-15

**Authors:** Jenny R. Nicolas, Zeyu Hui, Qiushi Miao, Haichen Lin, Michael R. Davidson, Ping Liu

**Affiliations:** ^a^Program in Materials Science and Engineering, University of California San Diego, La Jolla, CA 92093; ^b^Aiiso Yufeng Li Family Department of Nanoengineering, University of California San Diego, La Jolla, CA 92093; ^c^Department of Mechanical and Aerospace Engineering, University of California San Diego, La Jolla, CA 92093; ^d^School of Global Policy and Strategy, University of California San Diego, La Jolla, CA 92093

**Keywords:** lithium metal, battery, image analysis, morphology

## Abstract

Characterizing the morphology of lithium (Li) is crucial for developing long-lasting lithium metal batteries. It is well established that more uniform Li deposition correlates with better cell performance. Li morphology is often characterized through qualitative analysis of scanning electron microscopy (SEM) images; however, there are no widely accepted metrics to quantitatively describe deposition uniformity. Here, we propose a framework to quantify uniformity through SEM image analysis via the index of dispersion (*ID*) metric, which is defined and presented in the context of Li metal batteries. We also explore experimental impacts of sampling protocols on *ID* measurements. Our results demonstrate that the *ID* metric is highly sensitive to variations in deposition uniformity, including the coexistence and uniformity of multiple morphologies, uniformity within a single morphology, and particle size distribution uniformity. Furthermore, it is demonstrated that uniformity, as measured by the *ID*, can be related to the average potential of Li||Li symmetric cells over cycling. Higher capacity cycling leads to more pronounced changes in both *ID* and average cell potential. Local minima/maxima are found consistently in both *ID* and average cell potential immediately before cells short-circuit, which we suggest may indicate a collapse of the microstructure prior to failure. We put forward this framework as a more robust approach to quantify Li deposition uniformity, advancing the development of Li metal batteries that are safer and longer lasting.

## Significance

In this study, we present a simple yet effective method for quantitatively measuring the uniformity of lithium (Li) plating in lithium metal batteries using a traditionally qualitative technique, scanning electron microscopy. We examine how the uniformity, quantified through the index of dispersion (*ID*), evolves with cycle life. Our findings reveal that as cell cycling progresses, the nonuniformity or *ID* increases, accompanied by a rise in average cell potential. This innovative approach not only provides insights into cell failure through the *ID* metric but also allows for a more precise, quantitative analysis of Li morphology. Understanding how Li morphology changes over time is essential for tracking battery degradation, predicting lifespan, and optimizing cycling protocols, ultimately advancing the path toward commercialization.

Our societal need for higher energy density batteries necessitates research into lithium metal batteries (LMBs) due to their high theoretical capacity (3,861 mAh/g), approximately 10 times greater than that of typical lithium-ion battery (LIB) anodes (1). To commercialize the LMB system, safety and long cycle lifetimes are key. An understanding of what impacts battery life is critical, and among many others, lithium (Li) morphology has been identified as an important factor, since nonideal Li electrodeposition can lead to stochastic failure events known as short circuits. Typically, more uniform Li deposition results in better cell performance due to dendrite mitigation (2, 3). Dendrites are fingerlike Li projections growing from the anode to the cathode that puncture the separator layer, resulting in thermal runaway and causing premature cell failure (4, 5). Notably, these are point failure events (2). While traditional electrochemical techniques used for batteries effectively measure the ensemble cell behavior of Li plating and stripping, they forego nuanced details about Li morphology. Whereas, imaging techniques enable more local characterization critical for identifying these point failures (6, 7).

Quantitative imaging techniques such as operando neutron radiography (8), chemical shift imaging (9), and MRI (10) can detect the presence of Li with spatial resolution. However, despite their detailed insights into Li microstructure, they require highly specialized equipment and expertise. To generate large datasets with standard cell configurations (e.g., coin cell, pouch cell), quantitative metrics that leverage microscopy techniques are needed.

Foundational imaging work has used optical microscopy, scanning electron microscopy (SEM), transmission electron microscopy (TEM), and atomic force microscopy (AFM) to show Li morphology is an important factor in influencing cycling efficiency (6, 7). Yoshimatsu et al. used SEM images and Arakawa et al. used optical images to identify and understand failure in LMBs resulting from nonuniform Li deposition (11, 12). Similarly, the role of dendrites in LMB failures has been brought to light in other Li morphology studies (12–15). In conjunction with X-ray diffraction, SEM revealed that Li preferentially grows on certain crystal planes, highlighting crystallographic texture as a crucial parameter for achieving more uniform Li deposition (16, 17). While image analysis has been completed from the centimeter down to the nanometer length scale, there exists a knowledge gap regarding the length scale of Li uniformity that is most critical to cell lifetime. An optical image can capture an entire electrode, but resolution is limited. In contrast, TEM is typically used to capture phenomena at the nanometer to atomic level; however it has a significantly smaller field of view (7). SEM allows for spatial resolution from hundreds of microns to the submicrometer scale, capturing general surface morphology. However, current battery literature often relies on subjective comparisons of only a few microscopy images to assess uniformity, making its connections to the electrochemical performance less than robust. To reduce subjectivity, efforts have been made to develop quantitative metrics based on the width, height, and aspect ratio of Li particles to categorize Li morphology and assess nonuniform deposition (4). A crucial step toward objective analysis was made by binarizing SEM images to assess dead Li content but did not statistically evaluate uniformity or control for contrast levels across sampling conditions (18). Developing a comprehensive set of SEM-derived quantitative metrics is essential for accurately characterizing the uniformity of Li electrodeposits. Some recent efforts have begun exploring image analysis tools based on automation or machine learning, which demonstrate promising accuracy. These approaches often require synthetic training data, extensive preprocessing, and manual correction (19, 20). Furthermore, many studies focus on isolated snapshots and do not establish a framework to track morphological evolution across capacities or plating regimes.

Establishing quantitative uniformity metrics for LMBs requires addressing several challenges. These include image contrast and variability in particle shapes and sizes across different conditions. In SEM, topological contrast from the detection of secondary electrons from the sample encodes the size, shape, and texture of 3D objects in a 2D grayscale image (21). Making comparisons across electrochemical and imaging conditions is difficult. Key experimental considerations include whether to maintain a constant magnification or adjust the magnification to maintain a constant particle resolution between images, and how to implement objective image preprocessing steps. Bespoke software offers advanced segmentation tools; however, simple, accessible, and platform-agnostic tools are required for widespread adoption (22). Taking lessons from other fields, such as bioengineering, in which SEM is utilized to characterize particle size distributions (PSDs) (23, 24), sensitivity analyses are a common practice to account for the inherent subjectivity of the technique and to properly contextualize the uniformity metric for the given application (25–27). Similarly, the battery field must identify optimal imaging conditions through sensitivity analyses to ensure robust uniformity metrics.

Here, we present a comprehensive framework to quantify uniformity through a metric called the index of dispersion (*ID*). The *ID* describes the heterogeneity of lithium deposition, enabling rigorous and quantitative analysis of lithium morphology from SEM images. The *ID* is obtained through SEM image analysis using an open-source code, and a straightforward algorithm.

Our framework includes several key features to address the challenges discussed above: 1) metric selection: selecting *ID* as the primary metric and adapting it to our specific context; 2) pixel-based analysis: implementing a pixel-based approach that binarizes images and uses pixel counts to determine fractional coverage, ensuring consistent *ID* calculation independent of particle shape; 3) *ID* curve method: addressing contrast variability through a standardized thresholding process, with the highest resulting *ID* selected as the final *ID* value; and 4) parameter optimization: optimizing parameters such as image magnification and the number of slices through sensitivity analyses to balance precision and efficiency.

The theoretical basis for the *ID* is presented with careful consideration of all methodological choices to enable accurate and meaningful comparisons. We validate the approach using synthetic images with varying particle size distributions. We demonstrate this method’s capability to distinguish between different types of uniformity, including particle size distribution, the coexistence of multiple morphologies, and the regularity within a single morphology. Ultimately, we aim to establish a connection between *ID* and cycle life. These approaches seek to demonstrate the method’s broad applicability across diverse use cases.

## Results and Discussion

### Index of Dispersion Framework.

The *ID* has been used in many different fields including ecology, geology, and materials science (28). Kam et al. discusses the *ID* as a quality control metric for particle size distribution in nanocomposite manufacturing, as it is the most straightforward and widely recognized metric for calculating spatial uniformity (29). The *ID* measures uniformity based on the spatial distribution of particles. Each image is divided into a number of quadrats, *q*. The number of particles, $\left\{x_{1},x_{2},\cdots x_{q}\right\}$ are counted per quadrat. Next, the sample mean, $\bar{x}$, and sample of the counts, s, are calculated. Eq. **1** shows the formula for *ID* as according to Kam et al. (29).

[1] $$ID=\frac{(q-1)s^{2}}{\bar{x}},$$

$$\mathrm{where}\;\;s=\sqrt{\frac{\Sigma (x_{i}-\bar{x}{)}^{2}}{q}}.$$

If there is complete spatial randomness (CSR), there would be no particle clustering and an even distribution of particles per quadrat, with a low s resulting in a low *ID* value. If there is nonuniformity, there would be clustering of particles and an uneven distribution of particle counts per quadrat with a high s and a high *ID* value. In general, the higher the *ID* value, the further the distribution of counts are from CSR. A lower *ID* value implies a higher likelihood that the particles fall under CSR and reflects greater uniformity.

**Fig. 1.**
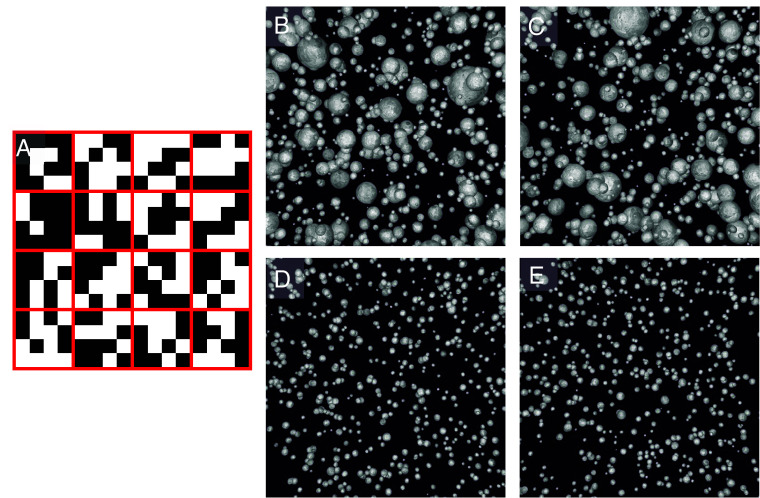
(*A*) Example image showing the total number of white pixels $\left(A_{white}\right)$ representative of the boundaries between Li particles, and the total number of pixels within each slice $\left(A_{\mathrm{total}}\right)$, divided into 16 slices (outlined in red) for the calculation of *ID*. Each of the 16 slices contains 16 pixels. (*B*–*E*) Synthetic SEM images of known PSDs used for *ID* calculation (30). (*B* and *C*) Lognormal particle size distribution, mean particle size of 0.12 and distribution shape parameter of 0.6. (*D* and *E*) Normal particle size distribution, mean particle size of 0.1 and distribution shape parameter of 0.025. These values are measured in arbitrary Blender units. Reproduced, Copyright 2016, Elsevier (30).

We opt to use this specific metric due to its simplicity instead of other quadrat-based metrics. However, the meaning of ${\{x}_{1},x_{2},\cdots x_{q}\}$ and $\bar{x}$ is adapted to represent the fractional coverage of Li (${\mathrm{FC}}_{i}$) and the mean fractional coverage of Li among all quadrats or slices (${\mathrm{FC}}_{\mathrm{avg}})$. In other words, the total number of white pixels ${(A}_{\mathrm{white}})$, representative of the boundaries between Li particles, is divided by the total number of pixels within each slice $\left(A_{\mathrm{total}}\right)$, and the average value is taken. This is illustrated in Fig. 1*A*.

With these definitions, Eq. **1** is adapted into Eq. **2** below. $\bar{x}$ is changed to ${\mathrm{FC}}_{\mathrm{avg}}$ and the equation for s is updated accordingly to reflect the uniformity of pixels or boundaries between Li particles rather than the number of particles:

[2] $$ID=\frac{(q-1)s^{2}}{{\mathrm{FC}}_{\mathrm{avg}}},$$

$$\mathrm{where}\;\;s=\sqrt{\frac{\Sigma ({\mathrm{FC}}_{i}-{\mathrm{FC}}_{\mathrm{avg}}{)}^{2}}{q}},\;{\mathrm{FC}}_{i}=\frac{A_{\mathrm{white}}}{A_{\mathrm{total}}}.$$

Additionally, a sensitivity study was conducted to determine the optimal number of quadrats or slices to use per image (*SI Appendix*, Figs. S1 and S2). The *SI Appendix* also includes a discussion on the optimal magnification.

Each SEM image receives its own *ID* value. For a given electrochemical condition, two replicate cells are made. Within each replicate cell, five images at random locations are acquired (diagram shown in *SI Appendix*, Fig. S3). The sample *ID* (*SID*) is calculated as the average *ID* across the total number of individual images ($P_{images}$) for each sample:

[3] $$SID=\frac{1}{P_{images}}*\sum_{1}^{P}{\mathrm{ID}}_{1}+{\mathrm{ID}}_{2}+{\mathrm{ID}}_{3}+\cdots +{\mathrm{ID}}_{P}.$$

The SID is taken as the final metric to use for a given electrochemical condition. It provides a more comprehensive view of the sample, considering each image as a component of the entire sample to obtain the average uniformity across the whole sample. As previously mentioned, the particle boundaries act as a proxy for particle size, the SID can give us a proxy for particle size dispersity.

The Global Uniformity (GU) metric is calculated as the SD across the entire sample of image *ID*s:

[4] $$GU=\sqrt{\frac{\Sigma ({\mathrm{ID}}_{i}-SID{)}^{2}}{P_{images}}},$$

where  i=1, 2, 3, ⋯, P.

The GU enables us to measure uniformity on a different length scale. While a single image may exhibit nonuniformity, the presence of consistent patterns across the sample as a whole indicates an underlying global uniformity. By considering that each SEM image at the chosen magnification (1000x) encapsulates 0.0288 mm^2^ of a 1.76 cm^2^ sample, the SID measures the average uniformity between all of the sample images. In contrast, the GU measures the *aggregate* uniformity of all of the sample images. Using 10 images per sample, the length scale has increased by a magnitude of 10 to 0.288 mm^2^. In this study, both the SID and GU are used to account for the potential influence of different length scales on cell lifetime.

### Application of *ID* Analysis to Synthetic Data.

To demonstrate the fidelity and application of the *ID* metric, we begin by using synthetic SEM images of powder materials with known ground truth PSDs. The synthetic images were created using Blender, an open-source 3D graphics suite used for modeling and scientific visualization (30, 31). Example images are shown in Fig. 1 with calculated *ID* values in Table 1. For our purposes, the white pixels are representative of the boundaries between Li particles and the black pixels are representative of a Cu substrate. Fig. 1 *B* and *C* are unique images made with the same lognormal particle size distribution with a mean particle size of 0.12 and distribution shape parameter of 0.6, while Fig. 1 *D* and *E* are unique images made with the same normal particle size distribution, with a mean particle size of 0.1 and distribution shape parameter of 0.025. These values are measured in arbitrary Blender units. Both PSD curves are shown in *SI Appendix*, Fig. S4. Due to their wider PSD, Fig. 1 *B* and *C* have higher ID values while Fig. 1 *D* and *E* have lower ID values, indicating greater uniformity which can be attributed to their narrower PSD. For each of the two pairs, the SID is calculated with the corresponding GU. The GU value for both Fig. 1 *B* and *C* and Fig. 1 *D* and *E* is either approximately or equal to zero, reflecting the consistency of dispersion patterns between the two image sets. The results for the full dataset of 2,048 synthetic SEM images of powder materials are shown in *SI Appendix*, Fig. S4 demonstrating good agreement between PSDs and *ID*.

**Table 1.** *ID* values for individual images and their representative sample

	Ground truth	*ID*	*SID*	*GU*
a	Lognormal Mean = 0.12, Shape = 0.6	0.37	0.36	0.01
b		0.35		
c	Normal Mean = 0.1, Shape = 0.025	0.15	0.15	0
d		0.15		

### Application of *ID* Analysis to Experimental Data.

In the example images used (Fig. 1 *B*–*E*), the same levels of contrast can be assumed across these images. However, grayscale micrographs obtained through SEM under different conditions will have different levels of contrast. In developing a robust method to account for varying levels of contrast between images, the *ID* based on different levels of binarization for each image is calculated. Binarization refers to converting a grayscale image into a binary image by assigning pixels a value of either 0 or 1 based on whether they fall below or above a specified threshold intensity pixel value.

**Fig. 2.**
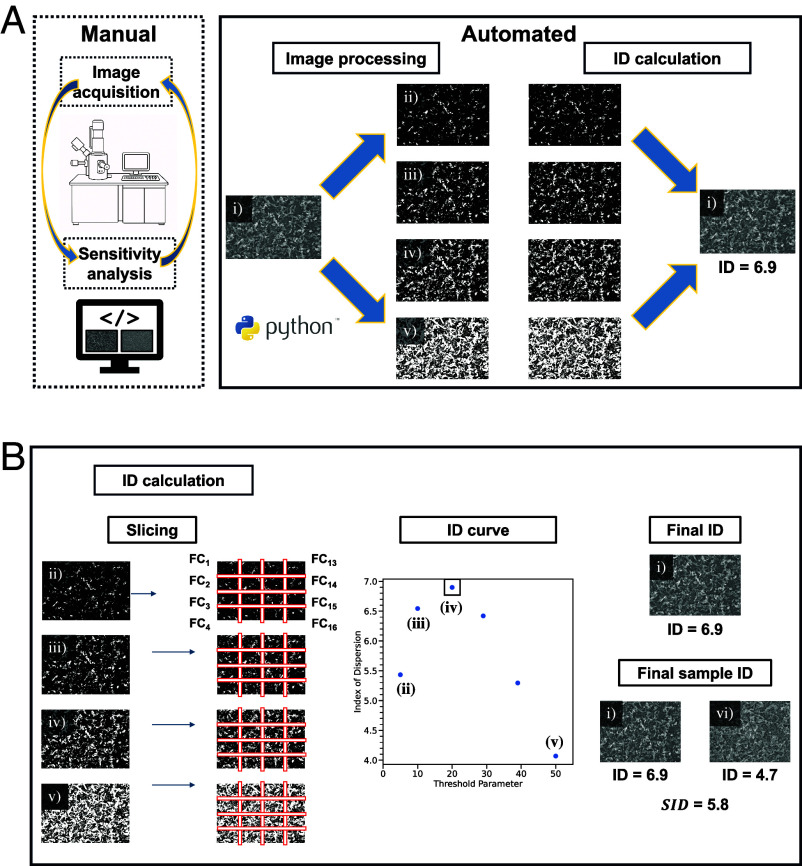
Workflow for *ID* calculation. (*A*) Image acquisition using SEM is followed by image processing using open-source python packages. In this step, (i) the original image of Li deposited onto Cu at low capacity is thresholded based on the top (ii) 5%, (iii) 10%, (iv) 20%, and (v) 50% brightest pixels to account for differences in contrast. The *ID* is calculated for each image as shown in *B*. (*B*) Each image is sliced into 16 quadrats and an *ID* for (ii–v) is calculated according to Eq. **2**. Each slice contains a unique fractional coverage, $FC_{i}$. *ID* is plotted as a function of threshold and the largest *ID* value is taken as the *ID* for the specific image (outlined point iv) to enable fairer comparisons between images. For example, 6.9 is the *ID* determined for image (i). Finally, the *SID* is obtained by averaging the *ID*s from images (i) and (vi), both from the same sample, according to Eq. **3**. Note, only the image acquisition and sensitivity analysis to determine the optimal magnification are manual steps (indicated by the dotted lines), while all other steps are automated (indicated by the solid lines).

For clarity the full workflow from image acquisition to final sample *ID* value is shown in Fig. 2. Image acquisition using SEM is followed by image processing using open-source python packages. The original image (Fig. 2 i) is binarized so that only the top 5% brightest pixels from the original image appear in Fig. 2 ii, top 10% brightest pixels in Fig. 2 iii, top 20% brightest pixels in Fig. 2 iv and top 50% in Fig. 2 v. An *ID* value is calculated for each thresholded image as outlined in Eq. **2** and shown in the Slicing component of Fig. 2*B*. An *ID* curve is constructed by plotting the *ID* as a function of the threshold parameter. While the *ID* varies with the binarized value or FC, the maximum *ID* reached is from the 20% thresholded image (Fig. 2 iv) so this is the *ID* assigned for Fig. 2 i. Finally, the IDs from individual images but taken from the same sample (Fig. 2 i and Fig. 2 vi) are averaged to obtain the (Eq. **3**).SID

In this way, each starting image can be equilibrated by different brightest pixel levels, removing the subjectivity of manually choosing threshold values, ensuring that all images undergo the same standardized process. Further, instead of applying an absolute pixel intensity threshold, which could be affected by variations in brightness and contrast across images, the method uses a percentage threshold to keep the proportion of brightest pixels constant across images. This guarantees that each image is processed consistently, regardless of its original brightness or contrast. This method allows for a comparison among different operating conditions which vary between SEM instruments and users. Aside from the image acquisition and sensitivity analysis to determine the optimal magnification, this entire procedure is automated by utilizing a python script with only the original images needed as input.

### Application of Method to Electrochemical Cells.

Our method is applied to calculate the *ID* for experimental data of Li deposited onto Cu at varying capacities and current densities to understand growth behavior of Li using a localized high-concentration electrolyte (LHCE) composed of 2 M Lithium bis(fluorosulfonyl) imide (LiFSI) in Dimethoxyethane (DME) and Bis(2,2,2-trifluoroethyl) ether (BTFE) in a 1:4 weight ratio (abbreviated as LDME). We used an LHCE system due to its high salt concentration that promotes an inorganic-rich solid–electrolyte interface (SEI). Its nonsolvating diluent reduces electrolyte viscosity and maintains the contact-ion-pair (CIP) structure which is more favorable to achieve good coulombic efficiency (32). Li metal batteries using an LHCE, however, suffer from premature shorting due in part to a lower ionic conductivity, resulting in nonuniformity of Li deposition (33, 34). Among other LHCEs, LDME has been shown to have a higher ionic conductivity and better Li deposition uniformity (2). In this work, LDME is selected as the electrolyte to measure the uniformity or lack thereof within a set of controlled cycling conditions.

**Fig. 3.**
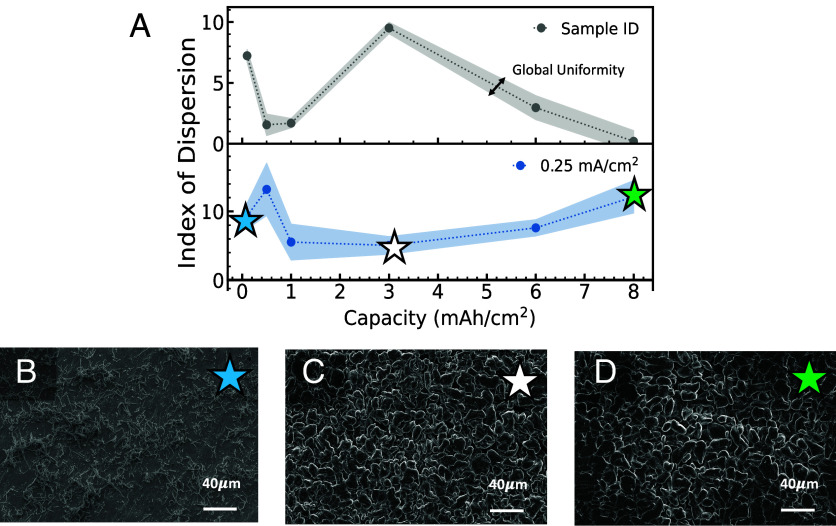
(*A*) *ID* plotted for Li deposited on Cu at 0.25 mA/cm^2^ with varying capacity conditions. The gray trace is shown as an example where the shaded region represents Global Uniformity. Ex situ SEM images of Li deposited on Cu at (*B*) 0.1, (*C*) 3, and (*D*) 8 mAh/cm^2^. Stars within each SEM image correspond to points in (*A*).

SEM images are collected during postmortem analysis after a single Li deposition step with different capacities (*SI Appendix*, Fig. S5*A*). The *ID* plotted for deposition capacities of 0.1, 0.5, 1, 3, 6, and 8 mAh/cm^2^ at a current density of 0.25 mA/cm^2^ is shown in Fig. 3*A*. At low capacities, as Li is plated onto Cu, the coexistence of Li growth and bare Cu is captured, as seen in Fig. 3*B* at 0.1 mAh/cm^2^. As the capacity is gradually increased, the Cu substrate is fully covered so the *ID* captures the nonuniformity within only one type of morphology, reflected by the minimum at 3 mAh/cm^2^ (Fig. 3*C*). At higher capacities, Li is deposited on top of Li and the particle size increases due to both the merging of particles and pressure effects, so the *ID* is a reflection of the particle size distribution. The wide particle size distribution at 8 mAh/cm^2^ is evident in Fig. 3*D*. The GU trends in Fig. 3*A* and *SI Appendix*, Fig. S5*B* follow a similar pattern as outlined for the *ID*, indicating that the trend in global uniformity is consistent with the trend in local uniformity. The gradual increase in Li capacity demonstrates that the method for *ID* calculation is highly sensitive in evaluating different types of uniformity: 1) between types of morphology, 2) within a certain morphological pattern, and 3) throughout the particle size distribution. This is also reflective of the regions delineated in Fig. 3*A* and shows the power of this algorithm to simultaneously discern between and quantify different types of uniformity relative to images of varying electrochemical conditions.

At a faster current density of 1 mA/cm^2^ for the same set of capacities, a consistent trend of IDs is obtained with the corresponding curve and sample images (*SI Appendix*, Fig. S5). The SIDs at this rate are lower (i.e., more uniform) than those at 0.25 mA/cm^2^. The dependence of Li nuclei size on current density has been studied. Using classical nucleation theory, it has been demonstrated that nuclei size is proportional to the inverse of overpotential (35). Plainly, the higher the current density, the smaller the nuclei size. In this case, SIDs are lower for 1 mA/cm^2^ compared to 0.25 mA/cm^2^ because of the magnification chosen. Due to the smaller particle size, at this length scale, the uniformity appears greater. If a higher magnification were chosen, there would be a less global view of the data and the 1 mA/cm^2^ data would no longer appear as uniform. This trend is consistently observed when comparing single deposition at these current densities; however, different behavior may emerge with repeated cycling.

### Relationship between Li Uniformity with Cell Cycling and Shorting Behavior.

**Fig. 4.**
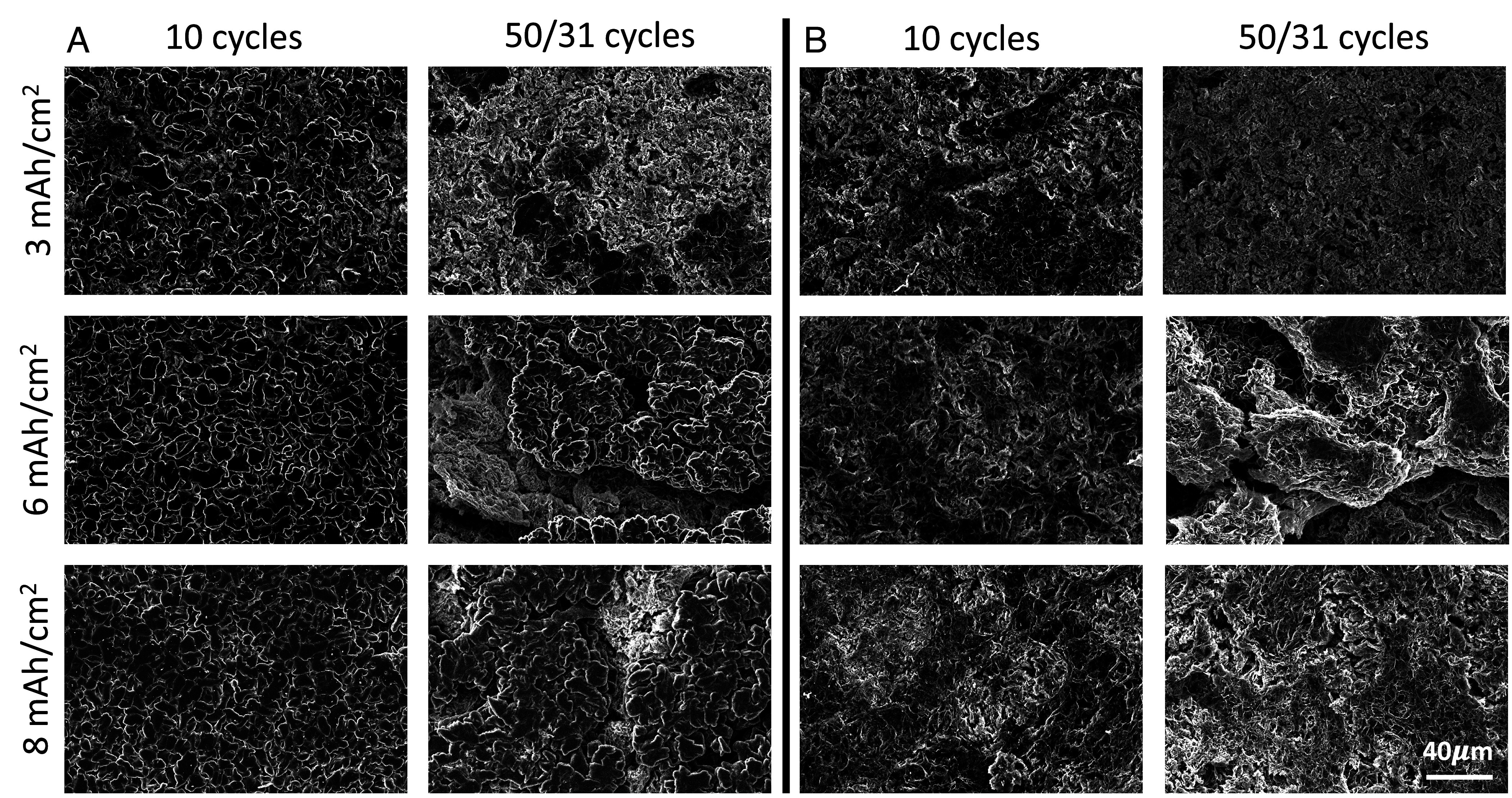
Ex situ SEM images from cycled Li||Li symmetric cells at 1 mA/cm^2^ with capacities of 3, 6, and 8 mAh/cm^2^ after cycles 10 and 50/31 of (*A*) plated Li and (*B*) stripped Li.

To investigate whether the *ID* can be used as an indicator for cell shorting behavior, the uniformity is probed as a function of increased cycling. Using symmetric Li||Li cells with LDME electrolyte, a current density of 1 mA/cm^2^ is selected along with capacity conditions of 3, 6, and 8 mAh/cm^2^. 3 mAh/cm^2^ was selected as the lowest capacity used in the Li||Li cell because it represents a practical and commonly studied value in the literature for lithium metal cells. This made it a natural reference point for studying how deposition uniformity evolves with cycling. For each capacity condition, approximately 100 images were acquired at varying magnifications. The Li morphology is analyzed on both the plated and stripped electrodes across the cycle life for each capacity condition, narrowing in right before the cell shorts to confirm whether there are significant changes in uniformity to cause shorting. The conditions used and images acquired are outlined in Table 2 and cell cycling data are shown in *SI Appendix*, Figs. S6–S8. As expected, the highest capacity cell of 8 mAh/cm^2^ shorts quickest after 35 to 36 cycles while the lowest capacity cell of 3 mAh/cm^2^ has the longest cycle life. SEM images were acquired after 1 and 10 cycles for all capacities used. To maintain a consistent comparison, we used 50 cycles for 3 and 6 mAh/cm^2^, and 31 cycles for 8 mAh/cm^2^ due to its shorter cycle life. Representative images of selected conditions are shown in Fig. 4 *A* and *B* with images from the other conditions shown in *SI Appendix*, Fig. S9.

**Table 2.** Electrochemical conditions upon which postmortem image analysis was completed

Capacity (mAh/cm^2^)	Cycle life	Cycle number
3	175 to 200	1, 10, 50, 100, 125, 150, 175
6	55 to 60	1, 10, 50, 55, 58
8	35 to 36	1, 10, 30, 31, 33

**Fig. 5.**
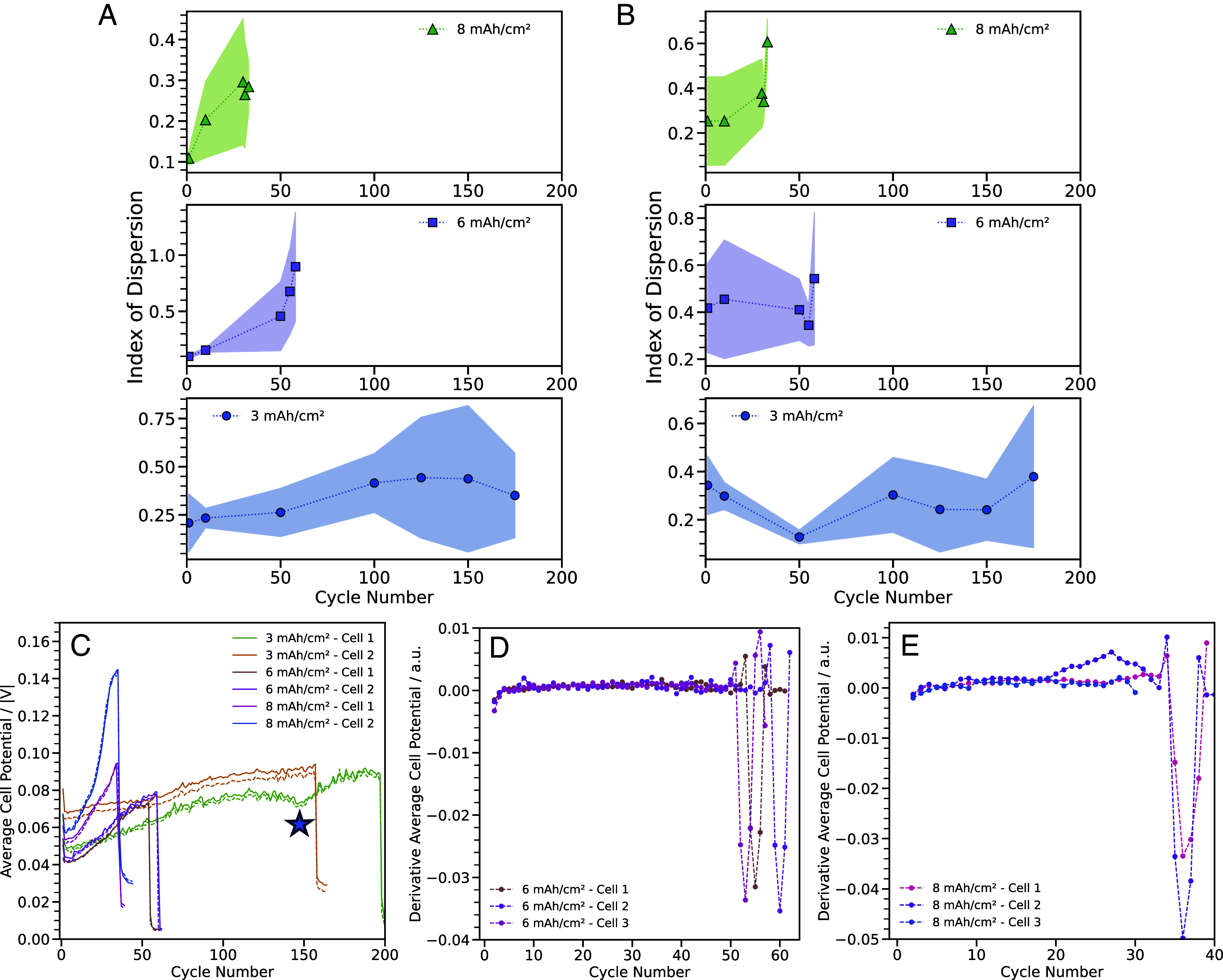
Change in *ID* with increased cycling for the varying capacity conditions from (*A*) plated Li images and (*B*) stripped Li images. (*C*) Plot showing the change in magnitude of average cell potential with increased cycling from Li||Li cells at 1 mA/cm^2^ with capacities of 3, 6, and 8 mAh/cm^2^. The average cell discharge potential is shown in the solid lines and the average cell charge potential is shown in the dotted lines. The blue star indicates the decrease in average cell potential for 3 mAh/cm^2^. Derivative of the average cell potential for cells at (*D*) 6 mAh/cm^2^ and (*E*) 8 mAh/cm^2^.

After 10 cycles, there is visually not much difference at the plated electrode. Differences in the uniformity of Li start to appear after 50/31 cycles. At 3 mAh/cm^2^, the representative SEM image shows mostly spongy Li with small chunks of denser Li. At 6 mAh/cm^2^, bigger chunks of denser Li are present with a smaller amount of spongy Li. At 8 mAh/cm^2^, there are even larger and flatter clumps of Li fused together. Fig. 5*A* shows the ID plotted against cycle number for the different capacity conditions. After the first cycle, the ID is similar for all capacity conditions. While the ID increases for all capacity conditions after 10 cycles, the values remain within a narrow range. The lines start to diverge with 6 and 8 mAh/cm^2^ having a greater slope indicating less uniformity of Li with cycling, compared to 3 mAh/cm^2^ which has a shallower slope with cycling. This is likely due to Li cycled at 6 mAh/cm^2^ containing the coexistence of chunky Li and spongy Li, which decreases its uniformity. While both 3 and 8 mAh/cm^2^ contain mostly one type of morphology, the chunky particles seen within 8 mAh/cm^2^ give rise to a smaller FC, reflecting a higher ID compared to the spongy particles from 3 mAh/cm^2^ with a higher FC and lower ID. Additionally, the GU indicated by the shading increases as a function of cycling for all capacity conditions. This indicates an increase in the global nonuniformity with cycling. Interestingly, all three capacities experience a change in trend of ID right before shorting, suggesting a change in local microstructure.

On the stripped side, there is a clear distinction between 3 mAh/cm^2^ and the higher capacities as shown in Fig. 5*B*. For the 3 mAh/cm^2^ condition, the maximum SID occurs after the first cycle and does not increase substantially over time. This may contribute to the improved long-term stability observed. In contrast, for the 6 and 8 mAh/cm^2^ conditions, the SID continues to grow and reaches its maximum value just before shorting—at 58 and 33 cycles, respectively. Further, there is a substantial difference between the GU at 3 mAh/cm^2^ and the higher capacities. The GU for 6 and 8 mAh/cm^2^ is large from the beginning, while it is much smaller for 3 mAh/cm^2^ and increases with cycling. The low degree of global uniformity seen on the stripped side in 6 and 8 mAh/cm^2^ is due to remaining SEI and dead Li. While dead lithium and SEI formation do occur at 3 mAh/cm^2^, they are not as pronounced as at higher capacities like 6 and 8 mAh/cm^2^. The 6 and 8 mAh/cm^2^ cells tend to short circuit earlier than the 3 mAh/cm^2^ cells, despite showing similar uniformity on the plated side at early cycles. However, differences in stripped-side uniformity suggest that early variations on the stripped electrode may drive long-term degradation. In particular, SEI growth and dead lithium buildup may increase porosity over time, leading to local current inhomogeneities that promote shorting. Therefore, even if the plated side appears uniform initially, the evolution of the stripped side plays a substantial role in overall cell stability.

Next, the electrochemical performance of the cycled cells is related with ID values. Taking representative cells from the three different capacity conditions, their average cell potential for both charge and discharge is plotted as a function of cycle number in Fig. 5*C*. The highest capacity condition has the largest gradient due to a multitude of factors. High areal capacity cycling leads to the formation of a thick and porous Li layer. The high surface area accelerates reaction with the electrolyte, leading to electrolyte depletion and formation of resistive side products. The tortuous paths in the porous Li also increases transport resistance. This type of porous structure can therefore lead to uneven Li deposition. This is consistent with the trend seen in ID on the plated side that the highest capacity condition has a higher gradient of ID. As shown in Fig. 4*A*, Li is plated on top of a porous structure which is the result of the side reaction between Li and the electrolyte. With a higher rate of cell degradation, this could likely lead to the emergence of greater nonuniformities of the microstructure with cycling. At the lowest capacity condition, the slope increases at a steadier rate. Again, this is consistent with the trend in ID that with cycling, the uniformity of plated Li decreases, though less drastically compared to the other capacity conditions.

Interestingly, features in average cell behavior before cell shorting are also observed. A decrease in average cell potential is seen before cycle 150 for 3 mAh/cm^2^ (marked by the blue star in Fig. 5*C*). This feature is also consistent with the trend in ID, in which the ID starts to level off around the 150th cycle and then starts to decrease. We conjecture that the decrease in average cell potential is due to a change in the Li microstructure. As the resistance and tortuosity of Li increases, the microstructure reaches a point of collapse due to the increased stack pressure in a coin cell. The decrease in cell overpotential leads to a partial recovery of the uniformity of the plated Li. Taking the derivative of the average cell potential, for cells at 6 mAh/cm^2^, peaks exist right before cell shorting, indicative of a rise in overpotential (Fig. 5*D*). The trend in ID is consistent as after 55 cycles, there is a sharp increase before cell shorting. Similarly, taking the derivative of the average cell potential for cells at 8 mAh/cm^2^, there is a steady decrease in overpotential from cycles 28 to 32, then an increase in average cell potential right before cell shorting (Fig. 5*E*). Again, this is consistent with the trend in ID in which there is a decrease in value at cycle 31, before the cell shorts.

Overall, a correlation has been established between trends in uniformity, as measured by the ID, and trends in cell potential. In cases of lower average cell potential, typically for cells cycling at lower capacity or earlier on in the cell’s cycle life, there is more uniform deposition. This manifests in a lower ID value on the plated side of Li in postmortem SEM analysis. However, as the cycle number increases, average cell potentials increase due to more morphological nonuniformities being introduced to the system, which is exacerbated at higher capacities. This results in lower levels of uniformity and is captured by an increase in ID value. The intent of this work is to provide practical parameters to guide cell design and better understand what levels of nonuniformity will result in cell shorting. This study serves as an initiative to quantify what these levels are, rather than relying solely on subjective views from postmortem image analysis. We show a representation of how this method can be used as a research tool for accelerated and quantitative postmortem cell diagnostics (*SI Appendix*, Fig. S10). While the focus of this work was to use the index of dispersion to study cell shorting, further investigation may use the index of dispersion to study the relationship between Li morphology and cell coulombic efficiency. Future work can also employ this method using cross-sectional instead of top-down SEM images to measure the uniformity of Li among layers and further track the evolution of morphology during growth.

This study advocates two advances in using SEM as an analytical tool for Li morphological analysis. The first is the need for many SEM images from different areas of an entire sample to represent the overall morphology, as opposed to drawing conclusions from only a few images. With the magnification used here, each image only represents 0.016% of the entire sample. It is critical to avoid sample bias so that the images chosen are indeed representative of the full sample. The second is to utilize SEM or optical microscopy images in conjunction with image analysis tools such as the ID metric, enabling a greater degree of reliability and more informative, quantitative findings.

## Conclusion

In summation, a framework and method were devised to measure the uniformity of Li via the ID metric. The power of this method is in its accessibility and simplicity by utilizing an open-source software with a straightforward algorithm. Varying levels of contrast across SEM images from different electrochemical conditions were accounted for by controlling for the level of thresholding. This method was validated within the context of Li deposition on Cu at various capacities which captured the method’s sensitivity in discerning between different types of morphology, within the morphology, and throughout the particle size distribution shown in an image. Postmortem image analysis was used as a prognosis tool to learn more about Li uniformity as it relates to cell life. We posit that a sample’s ID can be correlated with shorting behavior for this specific electrolyte system at varying capacity points at the rate of 1 mA/cm^2^. We hope in the future that researchers use this method to quantify Li uniformity for better comparison within the field in an effort to engineer Li metal batteries that are longer lasting. More broadly, we hope that this study will spur the battery field toward the use of other quantitative methods in image analysis.

## Materials and Methods

### Electrolyte Preparation.

The LDME electrolyte was prepared by dissolving 2 M LiFSI in DME/BTFE (1:4 w:w). Here, 1 M is defined as 1 M salt dissolved into 1 L of solvent. Lithium bis(fluorosulfonyl) imide (LiFSI) was purchased from Sigma-Aldrich. 1,2-Dimethoxyethane (DME) was purchased from Gotion, Inc. Bis(2,2,2-trifluoroethyl) ether (BTFE) was purchased from Tokyo Chemical Industry Co., Ltd. (TCI).

### Battery Assembly for Li Deposition Tests.

2016-type coin cells were used. Each cell included a 250 µm Li chip (15 mm in diameter), a 25 µm Celgard-2325 separator (19 mm in diameter), a piece of bare Cu (16 mm in diameter), two 0.5 mm spacers (15 mm in diameter) on either side of Li and Cu, and 60 µL of electrolyte.

### Battery Assembly for Li Shorting Tests.

2032-type coin cells were used. Each cell included two 250 µm Li chips (15 mm in diameter), a 25 µm Celgard-2325 separator (19 mm in diameter), two pieces of bare Cu (16 mm in diameter) between the Li and spacers. A 1.0 mm spacer (15 mm in diameter) and a spring were placed on the anode side under the Li. A 0.5 mm spacer (15 mm in diameter) was placed under the Li on the cathode side. 60 µL of electrolyte was used.

### Electrochemical Testing.

Li deposition and shorting tests were conducted on Neware and Land testers. For the Li deposition tests, a fixed amount of charge was passed galvanostatically at different current rates depending on the condition. For the shorting tests, a fixed amount of charge was passed galvanostatically at 1 mA/cm^2^.

### SEM Acquisition.

To harvest electrodes for SEM, cells were disassembled in an Ar-filled glovebox. The deposited Li was washed with DME to remove residual electrolyte. The uniformity of the deposited Li was characterized using SEM (FEI Quanta 250 Scanning Electron Microscope). In general, about 100 images were acquired for each sample condition at varying magnifications.

### SEM Information Content.

SEM measures topological contrast through the detection of secondary electrons generated by the interaction of the instrument’s beam of electrons with the atoms contained in a sample. The brighter parts of the micrograph typically represent particle edges or steeper surfaces as more secondary electrons are emitted from these parts of the sample due to a greater angle of incidence and a larger interaction volume (36). In this work, Li||Li cells were used to better understand Li shorting through the evolution of Li morphology, while we elected to use Li||Cu cells to develop the *ID* framework. The Cu substrate offers strong contrast, due to its denser electron cloud, enabling more effective characterization of Li deposition uniformity to refine and optimize the *ID* framework.

SEM images are taken of Li either on top of a Cu substrate or on top of existing layers of Li. In the case where Li is deposited on Cu, topological contrast is reflected by Li particles appearing brighter because the Li has been deposited directly on top of the Cu substrate. When Li is deposited on top of Li, the brighter areas are indicative of areas which are higher relative to the sample surface or of particle edges. As long as the substrate is fully covered with Li, the analysis should yield comparable results regardless of the initial substrate.

When binarizing images by assigning the brightest parts as white pixels, the ratio of particle perimeters to their area is measured. The aspect ratios of particles can be a proxy for particle size, while the particle–particle boundaries can be a proxy for particle size distributions.

### SEM Image Analysis.

Images were binarized in Python using OpenCV’s cv2.THRESH_BINARY method by retaining a fixed percentage of the brightest pixels in each image to account for contrast variations. For each thresholded image, the ID was calculated, and the maximum ID value was taken as the ID for the starting image. For each sample, two replicate samples were used and at least five spots within a sample were imaged for a more quantitative representation of the sample.

To obtain the ID value, the starting image was divided into 16 slices. For each slice, the total fractional coverage of Li was calculated (white pixels divided by the total number of pixels). The ID was calculated using information about the FC for each slice according to Eq. **3**.

Code for image analysis is available at https://github.com/jrnic123/Image_Analysis.

## Supplementary Material

Appendix 01 (PDF)

## Data Availability

The images used in this work, in addition to the code for implementing the image analysis are available at: https://github.com/jrnic123/Image_Analysis (37). Raw data are available upon request. All study data are included in the article and/or *SI Appendix*.
