# Supplementary material for: A quantitative imaging framework for lithium morphology: Linking deposition uniformity to cycle stability in lithium metal batteries
Source: Proc Natl Acad Sci U S A. 2025 Jul 15;122(29):e2502518122. doi: 10.1073/pnas.2502518122 (PMC12305041; doi:10.1073/pnas.2502518122)
Supplement: Supplementary file 1 — Appendix 01 (PDF) [file pnas.2502518122.sapp.pdf]

PNAS

Supporting Information for

A Quantitative Imaging Framework for Lithium Morphology: Linking Deposition Uniformity to Cycle Stability in Lithium Metal Batteries

Jenny R. Nicolas[1], Zeyu Hui[2], Qiushi Miao[1], Haichen Lin[1], Michael R. Davidson[3,4], Ping Liu[1,2*]

[1] Program in Materials Science and Engineering, University of California San Diego, La Jolla, CA 92093, United States

[2] Aiiso Yufeng Li Family Department of Nanoengineering, University of California San Diego, La Jolla, CA 92093, United States

[3] Department of Mechanical and Aerospace Engineering, University of California San Diego, La Jolla, CA 92093, United States

[4] School of Global Policy and Strategy, University of California San Diego, La Jolla, CA 92093, United States

* Ping Liu

Email: piliu@ucsd.edu

This file includes:

Figures S1 to S10

Sensitivity Study on Number of Slices for ID Calculation

A sensitivity study was conducted to determine the optimal number of quadrats or slices to use per image. Two SEM images of Li deposition on Cu substrate at the same capacity of 0.1 mAh/cm^2 but different current densities of 1 mA/cm^2 (***Fig. S1a***) and 0.25 mA/cm^2 (***Fig. S1b***) were compared, while two images from the same sample conditions of 1 mA/cm^2 and 6 mAh/cm^2 (***Fig. S1c-d***) were assessed. In comparing the two images from different conditions at a smaller number of slices, q, the ID for both samples start to converge at four slices since there is too little variance between each FC_i ***(Fig. S1e)***. For the images from the same sample condition which look similar, when using a greater q, the two images will have a different ID that is statistically significant at 128 slices as there is too large a variance between each FC_i ***(Fig. S1f)***. As a result, we need to compromise between these two extremes. It is important to ensure that the number of slices used contains a reliable amount of the image per slice. Sixteen slices per image are chosen to balance having a sufficient sample size of slices while maintaining a reliable amount of the image per slice. Each slice encompasses 6.25% of the image. The area of the largest particle included in our study amounted to 450 um^2, this means that at a minimum, each slice will contain four particles. If a greater number of slices were used, the number of particles appearing in each slice would be reduced.

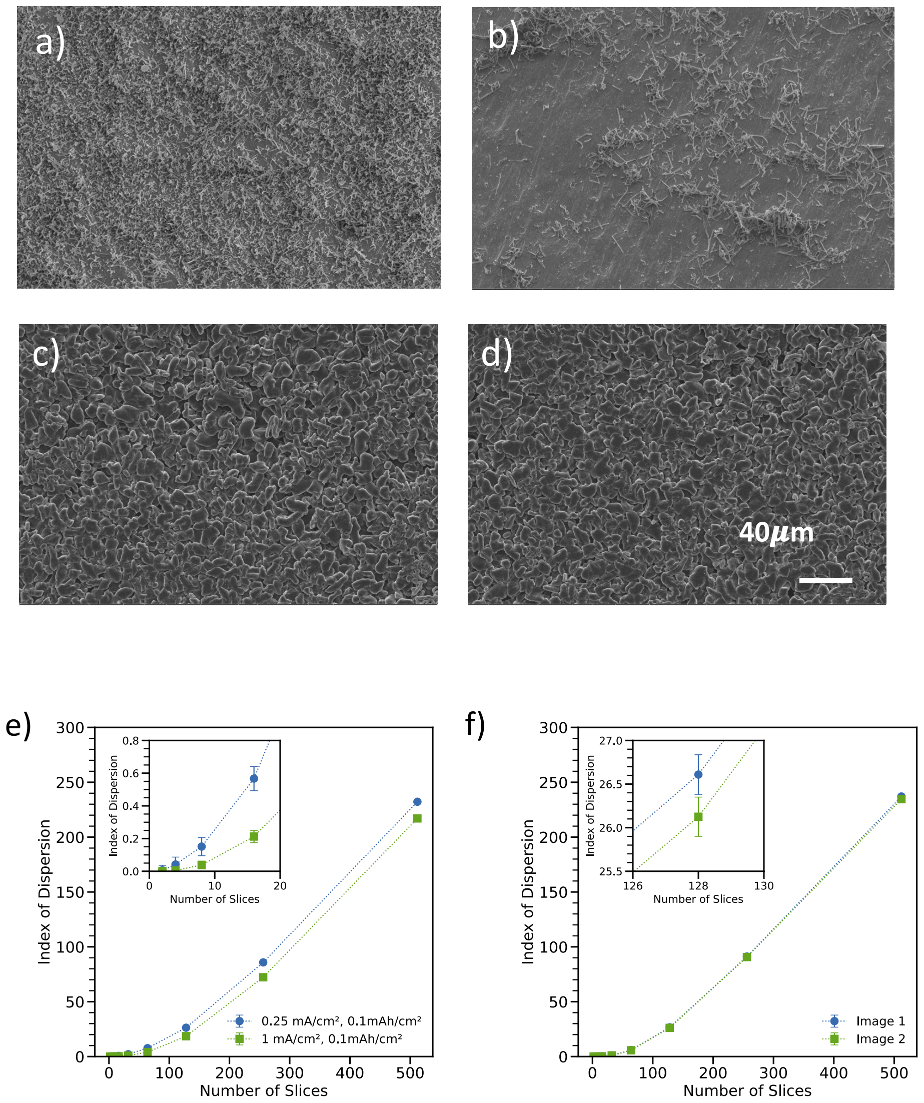

Figure S1. SEM images of Li deposition on Cu substrate at **a)** current density of 1 mA/cm^2 and capacity of 0.1 mAh/cm^2, **b)** current density of 0.25 mA/cm^2 and capacity of 0.1 mAh/cm^2, **c-d)** current density of 1 mA/cm^2 and capacity of 6 mAh/cm^2 from the same sample. Slice sensitivity analyses **e)** comparing image IDs at different conditions in (**b-c**) and **f)** comparing image IDs from the same sample condition in (**c-d**).

Study on Optimal Magnification for SEM Image Acquisition

One crucial consideration in determining how images from different electrochemical conditions will be compared is whether to maintain a constant magnification or to adjust the magnification in order to have a constant particle size. ***Fig. S2a*** shows ID values for Li deposited on Cu for 0.5 mAh/cm^2 at both 1 and 10 mA/cm^2 for varying magnifications. Images at the same magnification (1000x) for the low and high current density conditions are shown in ***Fig. S2b*** and ***d.*** It is evident that the particle size for each condition is very different. While individual Li particles are distinct in 1 mA/cm^2, at the higher current density it is more challenging to isolate individual particles as the much smaller particle size at this magnification makes particles look merged together creating the appearance of a more uniform surface. A comparable aspect ratio of the more noodle-like particles in the 1 mA/cm^2 sample at 1000x (***Fig. S2b***) is that of the particles in the 10 mA/cm^2 sample at 4000x (***Fig. S2e***). While a comparable aspect ratio of the chunkier particles in the 1 mA/cm^2 sample at 1000x is that of the particles in the 10 mA/cm^2 sample at 8000x (***Fig. S2f***). This presents the problem, that for each condition, there may be more than one type of particle shape or size, making it difficult to judge the size of representative particles, especially as particles start to merge. As a result, conditions are compared by controlling for the image magnification.

Fig. S2a serves as a sensitivity analysis to determine the optimal magnification with which to compare between electrochemical conditions. The ID values at 10 mA/cm^2 are unanimously lower than those for 1 mA/cm^2 no matter the magnification. This is due to both the effects of current density and subsequent spatial averaging. It is documented that the higher the current density, the smaller the particle size.[1] Additionally, materials composed of finer particles often exhibit greater apparent uniformity at larger length scales due to spatial averaging. As feature size decreases, local heterogeneities become less

visually or functionally prominent, leading to a smoother or more homogeneous macroscopic appearance.[2]

At low magnification (30x), large-scale inhomogeneities dominate the field of view, resulting in a highly non-uniform appearance. As magnification increases to 500x, individual particles become more resolved, and their spatial distribution appears more uniform, leading to a lower ID. However, beyond 1000x, the field of view becomes increasingly limited, and local variations become more pronounced, causing the ID to rise again. This trend highlights the breakdown of spatial averaging at high magnifications, where local heterogeneities outweigh the larger-scale uniformity. For this analysis, images of different conditions are compared at 1000x magnification to achieve an optimal balance between capturing microstructural nuances and retaining a meaningful level of information about the sample.

Currently, the magnification is chosen by a sensitivity analysis step which required manual supervision. In future, the algorithm can be automated for an objective comparison of uniformity without requiring uniform magnification. Moreover, the algorithm would evaluate the ratio of mean particle area to the total image area, ensuring that each image contains a statistically meaningful number of particles.

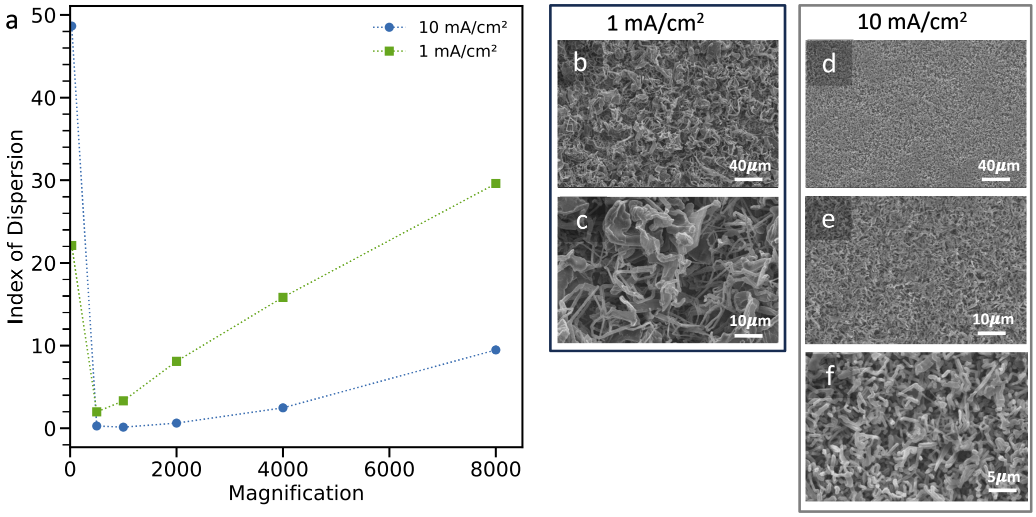

Figure S2. **a)** Graph of magnification and respective ID for 1 and 10 mA/cm^2. Ex situ SEM images of Li deposited on Cu at 1 mA/cm^2, 0.5mAh/cm^2 at a magnification of **b)** 1000x and **c)** 4000x. Ex situ SEM images of Li deposited on Cu at 10 mA/cm^2, 0.5mAh/cm^2 at a magnification of **d)** 1000x, **e)** 4000x, and **f)** 8000x.

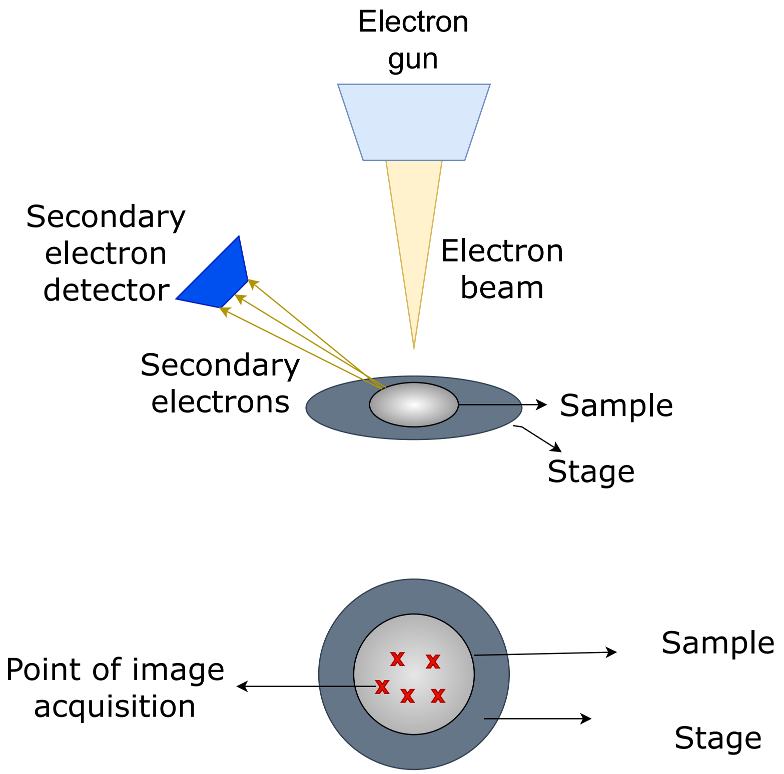

Figure S3. Diagram illustrating SEM sampling selecting five images at random locations within each cell.

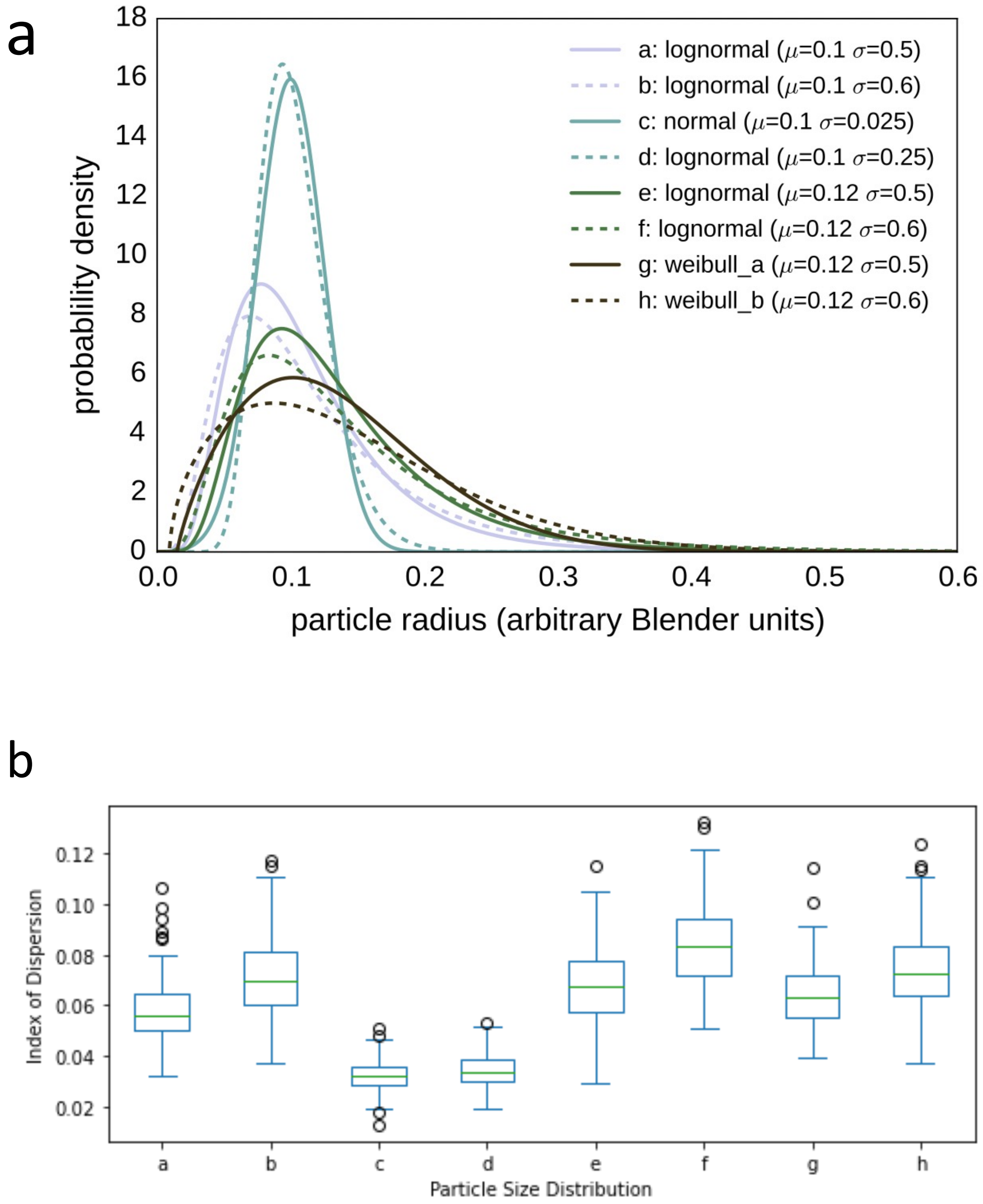

Figure S4. **a)** Distribution parameters for synthetic SEM images of known PSDs used for ID calculation. Unique images from PSDs *c* and *f* were used in the main text analysis as illustrative examples. Reproduced, Copyright 2016, Elsevier.[3] **b)** Box plots of ID for each PSD.

a

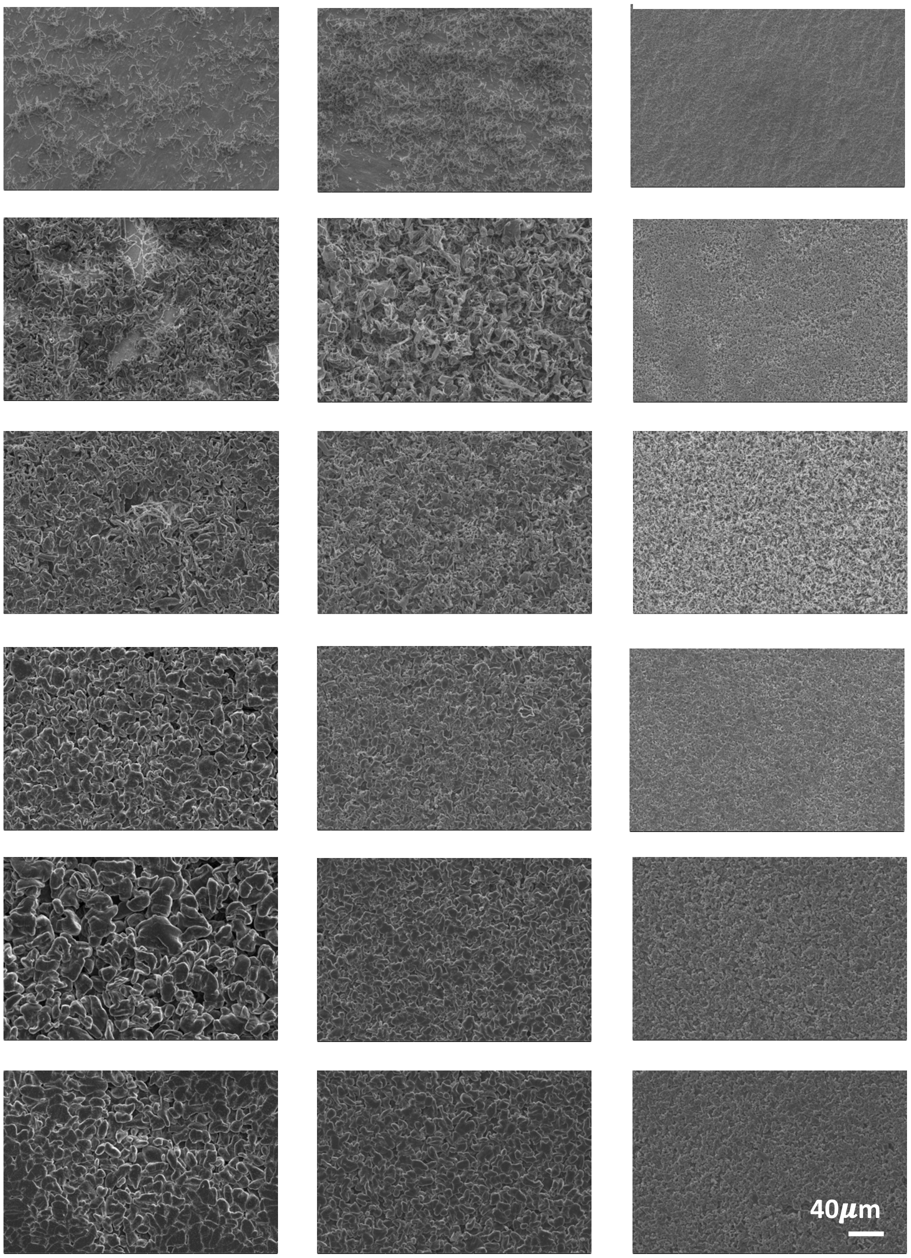
0.25 mA/cm2
1 mA/cm2
10 mA/cm2
0.1 mAh/cm2
0.5 mAh/cm2
1 mAh/cm2
3 mAh/cm2
6 mAh/cm2
8 mAh/cm2
40μm

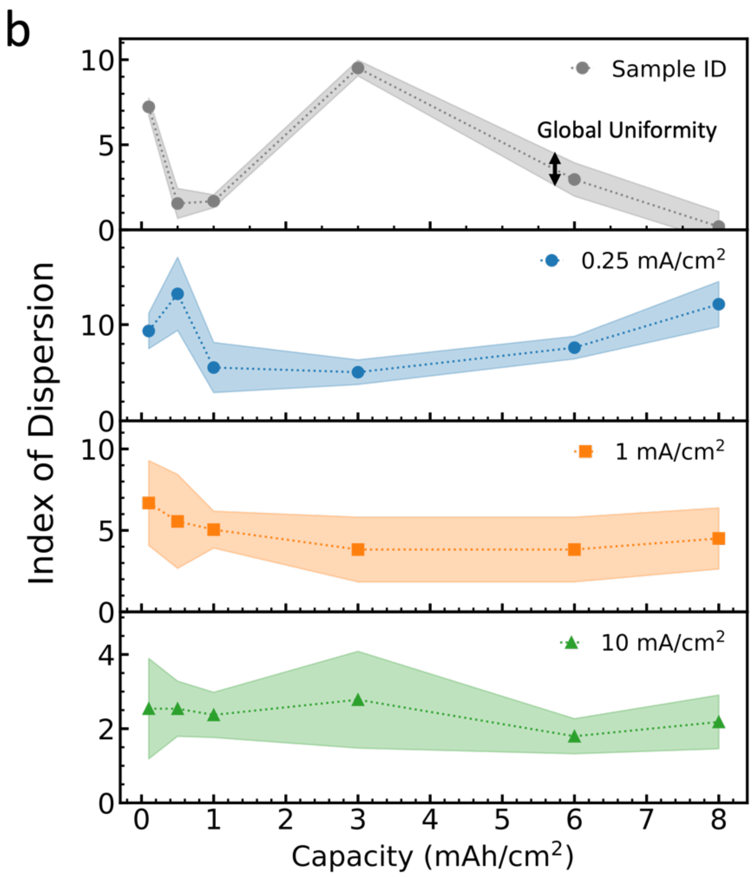

Figure S5. **a)** Ex situ SEM images of Li deposited on Cu at varying capacities and current densities. **b)** ID plotted for varying conditions. A gray trace is shown as an example where the shaded region represents Global Uniformity.

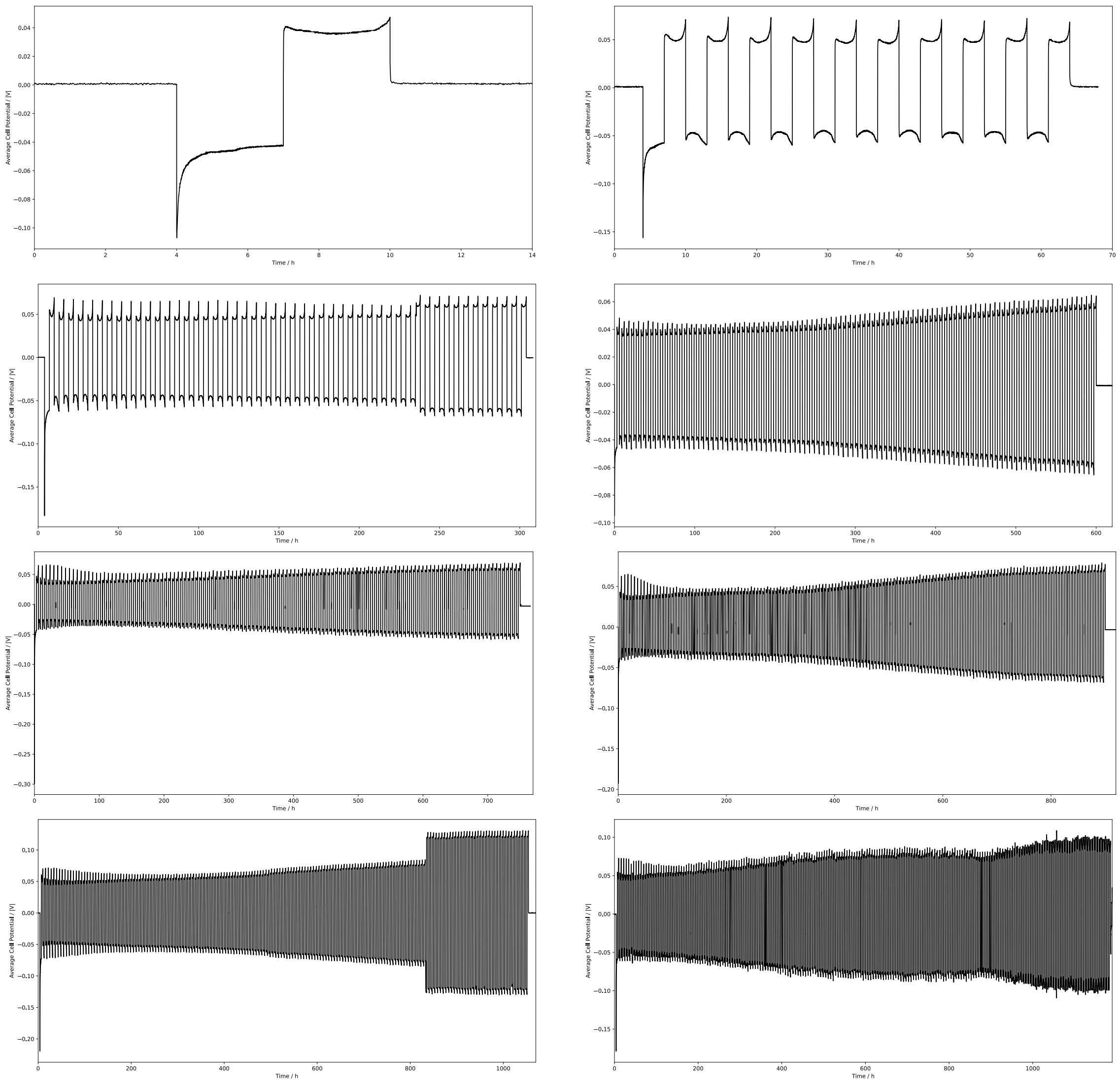

Figure S6. Cycling data for Li||Li, 1 mA/cm^2, 3 mAh/cm^2 after 1, 10, 50, 100, 125, 150, 175 cycles and until cell short circuits.

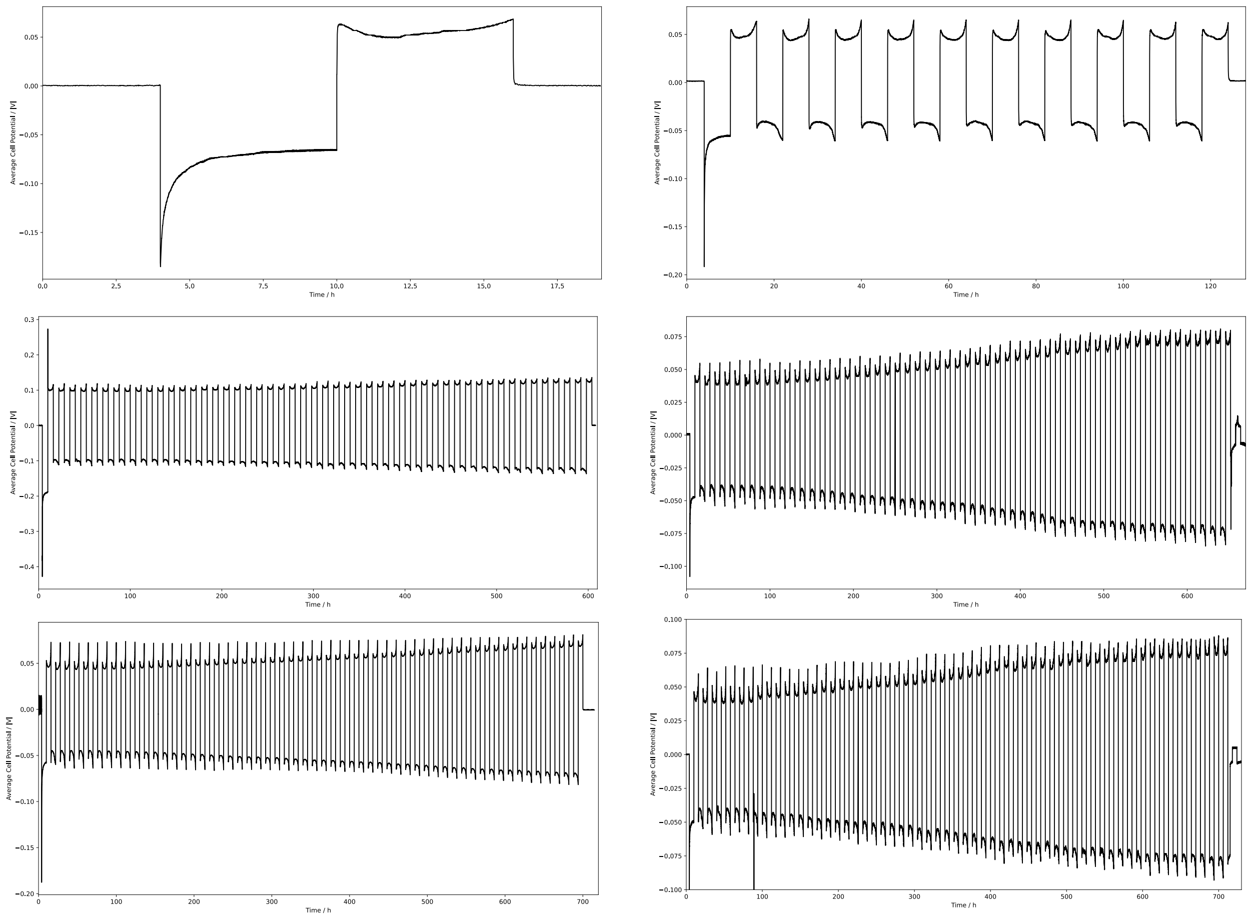

***Figure S7*.** Cycling data for Li||Li, 1 mA/cm^2, 6 mAh/cm^2 after 1, 10, 50, 55, 58 cycles and until cell short circuits.

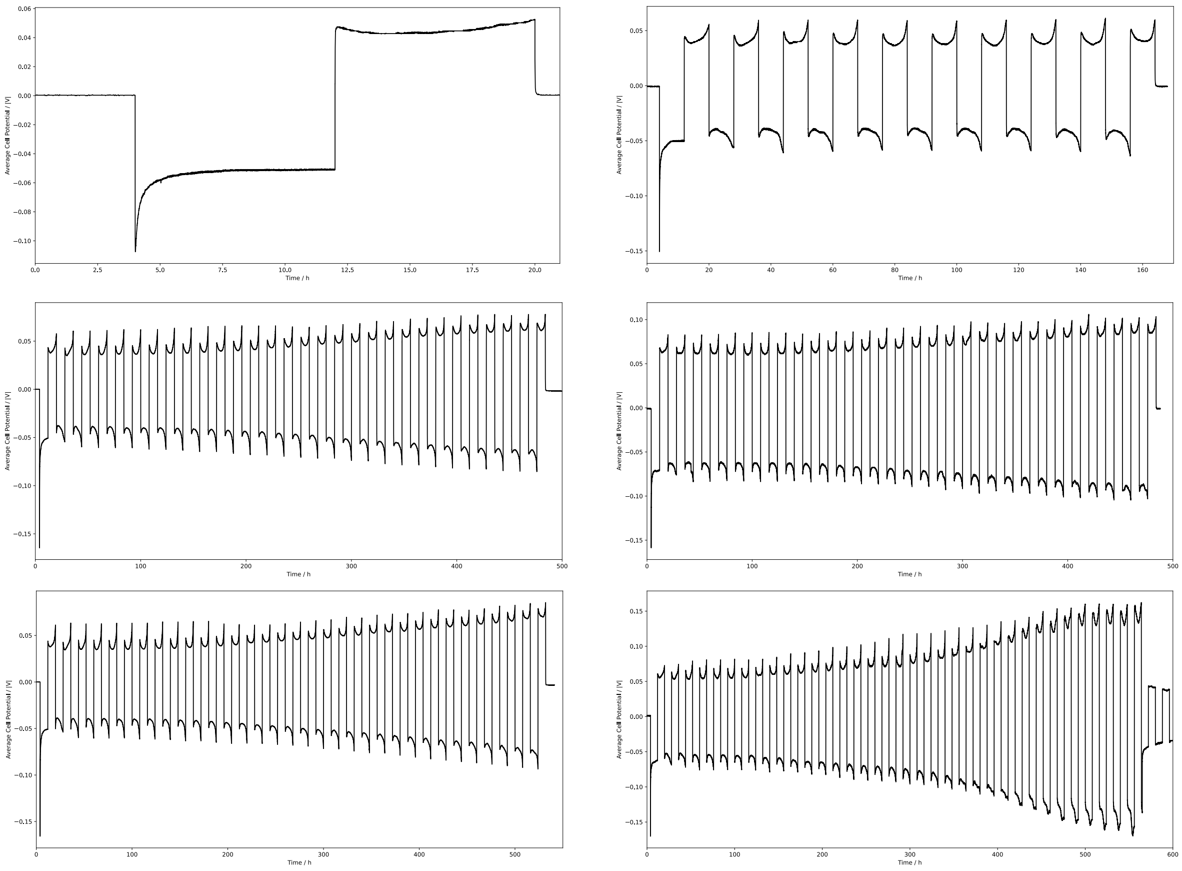

Figure S8. Cycling data for Li||Li, 1 mA/cm^2, 8 mAh/cm^2 after 1, 10, 30, 31, 33 cycles and until cell short circuits.

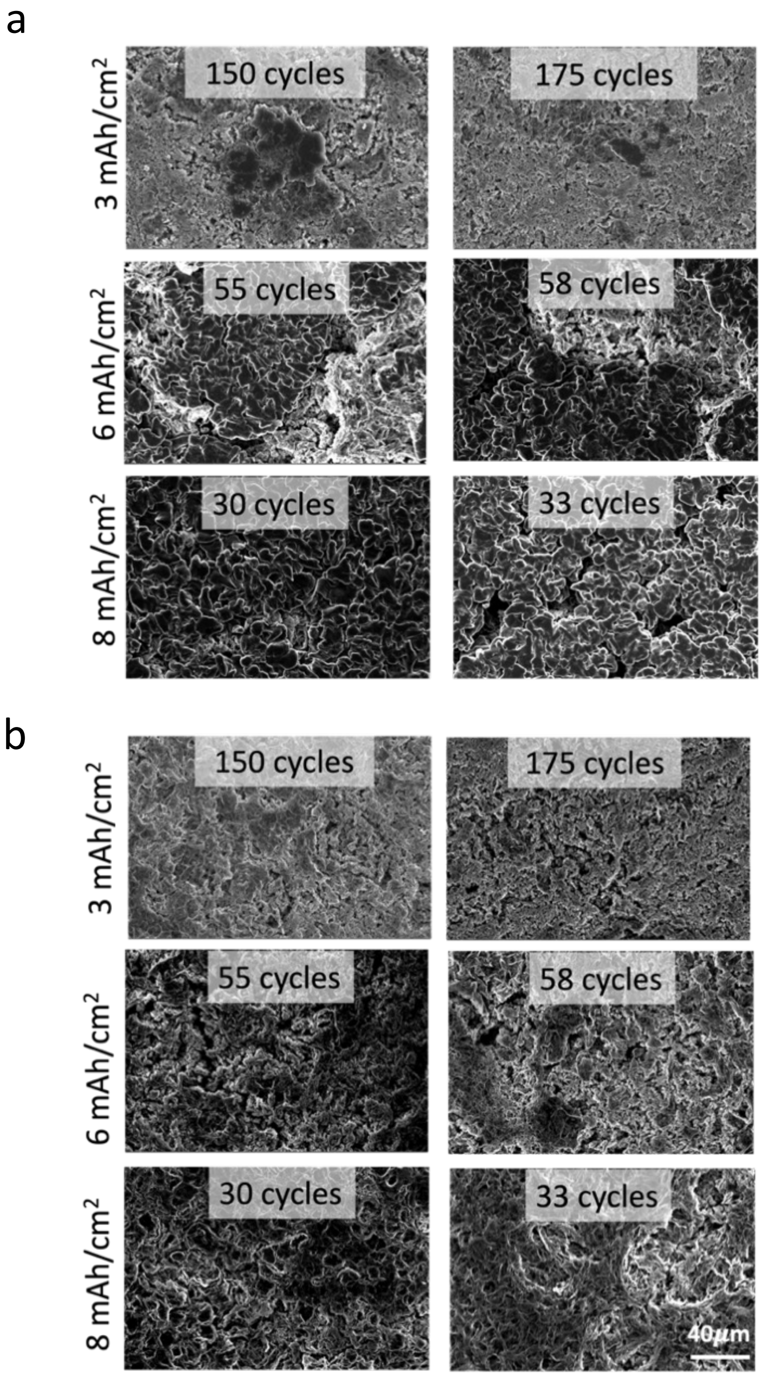

***Figure S9*.** Ex situ SEM images from cycled Li||Li symmetric cells at 1 mA/cm^2 with capacities of 3, 6, and 8 mAh/cm^2 of **a)** plated Li and **b)** stripped Li after cycles close to cell short circuiting.

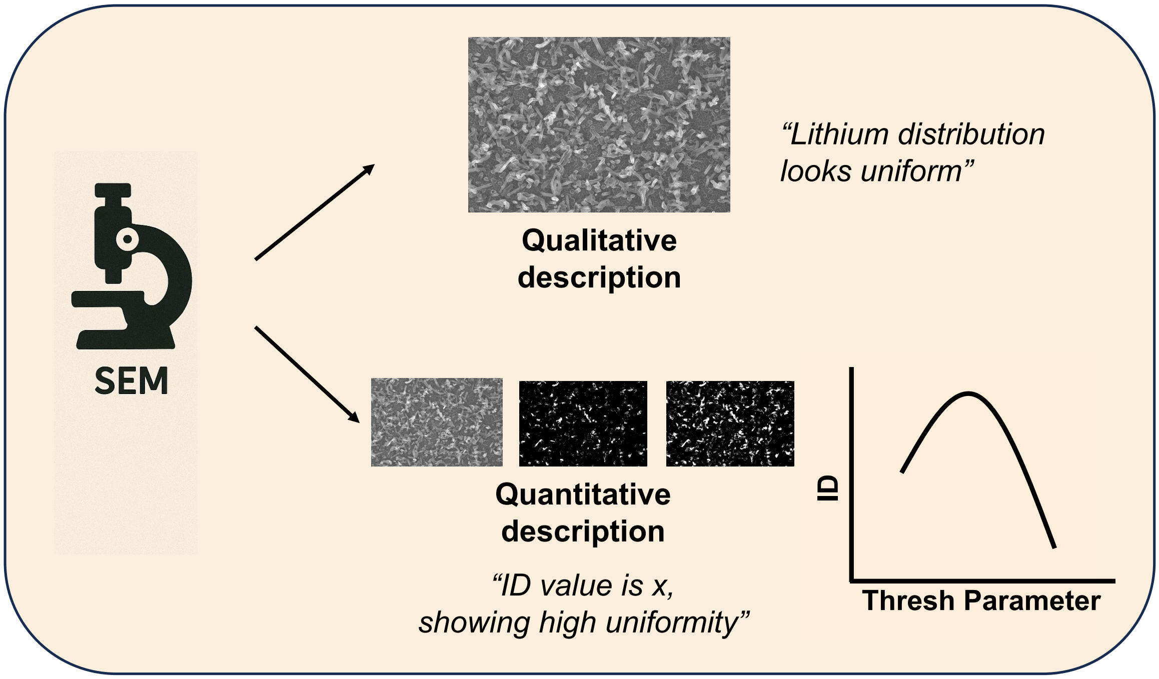

Figure S10. A schematic workflow showing how lithium deposition morphology, quantified via the ID can be used during the cell design and testing phase. After initial cycling, SEM imaging provides ID values that indicate whether a cell falls within a safe operating zone. This framework enables faster screening of cell designs and operating conditions without requiring full long-term cycling.

References

1. Pei, A., Zheng, G., Shi, F., Li, Y. & Cui, Y. Nanoscale Nucleation and Growth of Electrodeposited Lithium Metal. *Nano Lett.* **17**, 1132–1139 (2017).
2. Salvatore Torquato. *Random Heterogeneous Materials: Microstructure and Macroscopic Properties*. (Springer-Verlag, 2002).
3. DeCost, B. L. & Holm, E. A. A large dataset of synthetic SEM images of powder materials and their ground truth 3D structures. *Data in Brief* **9**, 727–731 (2016).
